# Supplementary material for: Evaluation and Modulation of Gut Microbiome Dysfunction in Chronically Critically Ill Patients: A Prospective Pilot Study
Source: Int J Mol Sci. 2025 Oct 8;26(19):9778. doi: 10.3390/ijms26199778 (PMC12525059; doi:10.3390/ijms26199778)
Supplement: Supplementary file 1 [file ijms-26-09778-s001.zip › ijms-3810056-supplementary.pdf]

Table S1. Antibiotics

Table S1. Drugs prescribed depending on the chosen treatment regimen.

	**Modulation regimen**		
Degree of dysfunction	Mild<br>n=21	Moderate<br>n=8	Severe<br>n=14
Drugs	Bactistatin n=17<br>Actoflor-S n=4	Bactistatin n=8<br>Doxycycline n=6<br>Nifuratel n=1<br>Amikacin n=1	Bactistatin n=9<br>Actoflor-S n=5<br>Doxycycline n=1<br>Clarithromycin, n=1<br>Amikacin n=4<br>Rifaximin n=2<br>Fosfomycin n=1<br>Ciprofloxacin, n=1<br>Cefepime n=2<br>Cefepime+Sulbactam n=2<br>Cefoperazone+ Sulbactam n =1<br>Meropenem n=4<br>Imipenem n =1<br>Vancomycin n=3<br>Linezolid n =1

Table S2. Dynamic monitoring of metabolites and biomarkers in patients (n=43) depending on the degree of microbiota dysfunction. The values <0.5 for AMM to samples with levels below the limit of quantification. The values <0.02 for PCT and <0.005 for S100 to samples with levels below the limit of quantification.

**Parameter**	**Mild (n=21)**	**Moderate (n=8)**	**Severe (n=14)**	**the Pearson chi-square or Kruskal-Wallis tests**
		**Day 1 of admission**		
**Metabolites**				
BA, μmol/L	0.9 (0.7;1.2)	0.7 (0.6;0.8)	0.8 (0.7;0.9)	0.145
PhLA, μmol/L	>0.5 (>0.5;0.5)	>0.5 (>0.5;0.5)	>0.5 (>0.5;0.5)	0.882
p-HBA, μmol/L	2.8 (>0.5;8.6)	0.8 (>0.5;2.0)	1.4 (1;2.5)	0.232
p-HPhAA, μmol/L	1 (>0.5;2)	0.9 (>0.5;2.2)	1.1 (0.5;3.0)	0.701
p-HPhLA, μmol/L	0.9 (0.6;1.2)	0.8 (0.6;1.2)	1.1 (1;1.3)	0.383
Σ3AMM, μmol/L	2 (1.5;3.4)	2.2 (1.3;4.2)	2.9 (1.8;4.2)	0.679
**Biomarkers**				
PCT, ng/ml	0.05 (0.03;0.11)	0.33 (0.07;2.57)	0.16 (0.12;0.26)	0.002<br>(**Gr**.1 vs **Gr**.2 0.048<br>**Gr**.1 vs **Gr**.3 0.005
CORT, nmol/L	348 (249;519)	757 (199;897)	596 (360;821)	0.322
IL-6, pg/ml	17.4<br>(12.9;43)	45.8 (19.2;87.4)	43.7<br>(31;59.8)	0.088
S100, mkg/L	0.08 (0.05;0.19)	0.05 (0.04;0.12)	0.1	0.602

			(0.06;0.15)	
NSE, ng/ml	12.9 (8.8;24.5)	7.4 (4.6;8.4)	12.3 (6;15.2)	0.155
**Day 7 of admission**				
**Metabolites**				
BA, µmol/L	0.9 (0.7;1.0)	0.7 (0.7;0.7)	0.9 (0.8;1.0)	0.235
PhLA, µmol/L	>0.5 (>0.5;0.5)	>0.5 (>0.5;0.5)	>0.5 (>0.5;0.5)	0.395
p-HBA, µmol/L	2.7 (>0.5;4.7)	1.1 (>0.5;1.2)	1.8 (1.3;3.1)	0.359
p-HPhAA, µmol/L	0.8 (0.5;2.6)	1.4 (0.7;2.3)	1.3 (0.8;2.6)	0.762
p-HPhLA, µmol/L	0.9 (0.6;1.1)	1.1 (1.0;1.4)	0.9 (0.7;1.3)	0.296
Σ3AMM, µmol/L	1.7 (1.3;4.0)	2.7 (1.9;4.4)	2.1 (1.8;4)	0.393
**Biomarkers**				
PCT, ng/ml	0.05 (0.04;0.14)	0.19 (0.06;0.38)	0.07 (0.04;0.12)	0.586
CORT, nmol/L	385 (268;565)	601 (492;731)	540 (492;599)	0.105
IL-6, pg/ml	14.7 (9.8;33.3)	46.7 (11.2;69.9)	31.1 (19.1;66.7)	0.125
S100, mkg/L	0.08 (0.05;0.14)	0.13 (0.04;0.3)	0.07 (0.05;0.16)	0.693
NSE, ng/ml	11.4 (6.9;22.0)	15.0 (11.1;19.1)	9.6 (5.4;17.6)	0.369
**Day 14 of admission**				
**Metabolites**				
BA, µmol/L	0.8 (0.7;1.0)	0.8 (0.7;0.9)	0.9 (0.7;1.4)	0.745
PhLA, µmol/L	>0.5 (>0.5;0.5)	>0.5 (>0.5;0.5)	>0.5 (>0.5;0.5)	0.437
p-HBA, µmol/L	0.6 (>0.5;4.51)	1.9 (1.7;2.1)	2 (1.6;3.5)	0.583
p-HPhAA, µmol/L	0.7 (>0.5;1.7)	1.4 (1;2.4)	1 (0.8;1.7)	0.145
p-HPhLA, µmol/L	0.7 (0.6;0.9)	0.8 (0.7;1)	0.9 (0.8;1.1)	0.319
Σ3AMM, µmol/L	1.4 (1.2;2.5)	3 (1.9;3.6)	2.1 (1.9;2.8)	0.094
**Biomarkers**				
PCT, ng/ml	0.1 (0.04;0.17)	0.04 (0.04;0.08)	0.1 (<0.02;0.1)	0.791
CORT, nmol/L	383 (294;623)	485.7 (385;669)	478.9 (449;566)	0.229
IL-6, pg/ml	20.7 (7.3;47)	18 (10.2;27.1)	23.7 (17.4;46.9)	0.75
S100, mkg/L	0.08 (0.03;0.15)	0.05 (0.02;0.09)	<0.005 (<0.005;0.1)	0.629
NSE, ng/ml	18.7 (9.1;21.7)	10.2 (7;14.2)	15.8 (5.1;24.7)	0.132

Table S3. Dynamic monitoring of microorganisms in patients (n=43).

Parametres	1 days	14 days	p-value
Bacterial mass, CFU	N = 21, Me = 1,0*10^12 [3,0*10^10; 9,0*10^12], m = 8,6*10^12 (2,0*10^13)	N = 21, Me = 3,0*10^11 [1,0*10^10; 4,5*10^12], m = 5,5*10^12 (1,3*10^13)	0,541
Lactobacillus spp., CFU	N = 21, Me = 1,0*10^5 [0; 8,5*10^5], m = 4,5*10^7 (1,6*10^8)	N = 21, Me = 1,0*10^5 [0; 1,0*10^5], m = 1,4*10^8 (6,5*10^8)	0,250
Bifidobacterium spp., CFU	N = 21, Me = 7,0*10^8 [5,0*10^7; 8,0*10^9], m = 4,2*10^10 (1,0*10^11)	N = 21, Me = 4,0*10^8 [5,0*10^7; 2,0*10^9], m = 9,4*10^9 (2,3*10^10)	0,338
Escherichia coli, CFU	N = 21, Me = 3,0*10^8 [3,5*10^7; 2,5*10^9], m = 2,0*10^9 (3,5*10^9)	N = 21, Me = 4,0*10^8 [2,0*10^7; 2,5*10^9], m = 2,2*10^9 (4,6*10^9)	0,926

Bacteroides spp., CFU	N = 21, Me = 1,0*10^12 [4,5*10^9; 9,0*10^12], m = 8,6*10^12 (2,0*10^13)	N = 21, Me = 2,0*10^11 [2,5*10^9; 4,5*10^12], m = 5,5*10^12 (1,3*10^13)	0,541
Faecalibacterium prausnitzii, CFU	N = 21, Me = 9,0*10^7 [2,0*10^6; 5,0*10^9], m = 1,2*10^11 (4,4*10^11)	N = 21, Me = 1,0*10^7 [0; 6,0*10^8], m = 4,3*10^10 (1,7*10^11)	0,233
Klebsiella pneumoniae, CFU	N = 21, Me = 0 [0; 0], m = 4,7*10^5 (2,0*10^6)	N = 21, Me = 0 [0; 0], m = 2,4*10^5 (7,7*10^5)	0,844
Klebsiella oxytoca, CFU	N = 21, Me = 0 [0; 0], m = 9,5*10^4 (4,4*10^5)	N = 21, Me = 0 [0; 0], m = 0 (0)	>0,999
Candida spp., CFU	N = 21, Me = 0 [0; 0], m = 2,4*10^6 (6,2*10^6)	N = 21, Me = 0 [0; 4,0*10^5], m = 7,5*10^6 (1,9*10^7)	0,570
Staphylococcus aureus, CFU	N = 21, Me = 0 [0; 4,0*10^7], m = 9,5*10^12 (4,4*10^13)	N = 21, Me = 0 [0; 5,0*10^5], m = 9,6*10^8 (4,4*10^9)	0,617
Escherichia coli enteropathogenic, CFU	N = 21, Me = 0 [0; 0], m = 1,9*10^3 (8,7*10^3)	N = 21, Me = 0 [0; 0], m = 0 (0)	>0,999
Enterococcus spp., CFU	N = 21, Me = 0 [0; 1,5*10^5], m = 2,0*10^6 (8,7*10^6)	N = 21, Me = 0 [0; 1,5*10^5], m = 9,5*10^7 (4,4*10^8)	>0,999
Bacteroides thetaiotaomicron, CFU	N = 20, Me = 1,5*10^8 [1,0*10^5; 7,8*10^8], m = 1,7*10^10 (6,7*10^10)	N = 20, Me = 0 [0; 1,0*10^9], m = 4,0*10^10 (1,8*10^11)	0,821
Akkermansia muciniphila, CFU	N = 21, Me = 0 [0; 1,0*10^6], m = 6,7*10^8 (2,2*10^9)	N = 21, Me = 0 [0; 0], m = 2,4*10^8 (7,7*10^8)	0,781
Clostridium difficile, CFU	N = 21, Me = 0 [0; 2,2*10^7], m = 1,1*10^9 (4,4*10^9)	N = 21, Me = 0 [0; 0], m = 3,4*10^9 (1,5*10^10)	0,813
Clostridium perfringens, CFU	N = 21, Me = 0 [0; 0], m = 2,9*10^7 (1,3*10^8)	N = 21, Me = 0 [0; 0], m = 0 (0)	0,500
Proteus spp., CFU	N = 21, Me = 2,0*10^8 [0; 4,5*10^10], m = 6,9*10^10 (1,6*10^11)	N = 21, Me = 1,0*10^9 [7,0*10^6; 7,5*10^9], m = 1,6*10^10 (4,5*10^10)	0,469
Enterobacter spp. / Citrobacter spp., CFU	N = 21, Me = 1,0*10^7 [0; 1,5*10^9], m = 2,4*10^9 (6,7*10^9)	N = 21, Me = 8,0*10^6 [0; 3,0*10^9], m = 2,3*10^9 (5,0*10^9)	0,670
Fusobacterium nucleatum, CFU	N = 21, Me = 0 [0; 0], m = 1,6*10^5 (6,6*10^5)	N = 21, Me = 0 [0; 0], m = 0 (0)	0,500
Parvimonas micra, CFU	N = 21, Me = 0 [0; 0], m = 5,3*10^6 (2,2*10^7)	N = 21, Me = 0 [0; 0], m = 4,8*10^5 (2,2*10^6)	0,250
Bacteroides fragilis group/ Faecalibacterium prausnitzii	N = 21, Me = 1,1*10^4 [5,2*10^1; 5,0*10^9], m = 2,4*10^9 (4,4*10^9)	N = 21, Me = 1,0*10^4 [7,3*10^2; 1,0*10^10], m = 2,9*10^9 (4,6*10^9)	0,985

Table S4. Data on monitoring clinical and laboratory parameters in patients depending on the degree of microbiota dysfunction, data are presented as median and interquartile range. The values <0.5 for AMM to samples with levels below the limit of quantification. The values <0.02 for procalcitonin (PCT) and <0.005 for S100 to samples with levels below the limit of quantification.

Parameter	Mild (n=21)	Moderate (n=8)	Severe (n=14)	the Pearson chi-square or Kruskal-Wallis tests
		Day 1 of admission		
**Metabolites**				
BA, μmol/L	0.9 (0.7;1.2)	0.7 (0.6;0.8)	0.8 (0.7;0.9)	0.145

PhLA, µmol/L	>0.5 (>0.5;0.5)	>0.5 (>0.5;0.5)	>0.5 (>0.5;0.5)	0.882
p-HBA, µmol/L	2.8 (>0.5;8.6)	0.8 (>0.5;2)	1.4 (1;2.5)	0.232
p-HPhAA, µmol/L	1 (>0.5;2)	0.9 (>0.5;2.2)	1.1 (0.5;3.0)	0.701
p-HPhLA, µmol/L	0.9 (0.6;1.2)	0.8 (0.6;1.2)	1.1 (1;1.3)	0.383
Σ3AMM, µmol/L	2 (1.5;3.4)	2.2 (1.3;4.2)	2.9 (1.8;4.2)	0.679
**Biomarkers**				
PCT, ng/ml	0.05 (0.03;0.11)	0.33 (0.07;2.57)	0.16 (0.12;0.26)	0.002 (Gr.1 vs Gr.2 0.048 Gr.1 vs Gr.3 0.005
CORT, nmol/L	348 (249;519)	757 (199;897)	596 (360;821)	0.322
IL-6, pg/ml	17.4 (12.9;43.9)	45.8 (19.2;87.4)	43.7(31;59.8)	0.088
S100, mkg/L	0.08 (0.05;0.19)	0.05 (0.04;0.12)	0.1 (0.06;0.15)	0.602
NSE, ng/ml	12.9 (8.8;24.5)	7.4 (4.6;8.4)	12.3 (6;15.2)	0.155
		**Day 7 of admission**		
**Metabolites**				
BA, µmol/L	0.9 (0.7;1)	0.7 (0.7;0.7)	0.9 (0.8;1)	0.235
PhLA, µmol/L	>0.5 (>0.5;0.5)	>0.5 (>0.5;0.5)	>0.5 (>0.5;0.5)	0.395
p-HBA, µmol/L	2.7 (>0.5;4.7)	1.1 (>0.5;1.2)	1.8 (1.3;3.1)	0.359
p-HPhAA, µmol/L	0.8 (0.5;2.6)	1.4 (0.7;2.3)	1.3 (0.8;2.6)	0.762
p-HPhLA, µmol/L	0.9 (0.6;1.1)	1.1 (1.;1.4)	0.9 (0.7;1.3)	0.296
Σ3AMM, µmol/L	1.7 (1.3;4.0)	2.7 (1.9;4.4)	2.1 (1.8;4)	0.393
**Biomarkers**				
PCT, ng/ml	0.05 (0.04;0.14)	0.19 (0.06;0.38)	0.07 (0.04;0.12)	0.586
CORT, nmol/L	385 (268;565)	601 (492;731)	540 (492;599)	0.105
IL-6, pg/ml	14.7 (9.8;33.3)	46.7 (11.2;69.9)	31.1 (19.1;66.7)	0.125
S100, mkg/L	0.08 (0.05;0.14)	0.13 (0.04;0.3)	0.07 (0.05;0.16)	0.693
NSE, ng/ml	11.4 (6.9;22)	15. (11.1;19.1)	9.6 (5.4;17.6)	0.369
		**Day 14 of admission**		
**Metabolites**				
BA, µmol/L	0.8 (0.7;1)	0.8 (0.7;0.9)	0.9 (0.7;1.4)	0.745
PhLA, µmol/L	>0.5 (>0.5;0.5)	>0.5 (>0.5;0.5)	>0.5 (>0.5;0.5)	0.437
p-HBA, µmol/L	0.6 (>0.5;4.51)	1.9 (1.7;2.1)	2 (1.6;3.5)	0.583
p-HPhAA, µmol/L	0.7 (>0.5;1.69)	1.4 (1;2.4)	1 (0.8;1.7)	0.145
p-HPhLA, µmol/L	0.7 (0.6;0.9)	0.8 (0.7;1)	0.9 (0.8;1.1)	0.319
Σ3AMM, µmol/L	1.4 (1.2;2.5)	3 (1.9;3.6)	2.1 (1.9;2.8)	0.094
**Biomarkers**				
PCT, ng/ml	0.1 (0.04;0.17)	0.04 (0.04;0.08)	0.1 (<0.02 ;0.1)	0.791
CORT, nmol/L	383 (294;623)	485.7 (385;669)	478.9 (449;566)	0.229
IL-6, pg/ml	20.7 (7.3;47)	18.(10.2;27.1)	23.7 (17.4;47)	0.75
S100, mkg/L	0.08 (0.03;0.15)	0.05 (0.02;0.09)	<0.005 (<0.005;0.1)	0.629
NSE, ng/ml	18.7 (9.1;21.7)	10.2 (7;14.2)	15.8 (5.1;24.7)	0.132

Parametres	*Group 1 (N = 18)*	*Group 2 (N = 4)*	*Group 3 (N = 11)*	*p-value 1vs2vs3*	*p-value 1vs2*	*p-value 1vs3*	*p-value 2vs3*
Days 1							
p5#1 сутки#Общая бактериальная масса, КОЕ	*N = 14, Me = 5,0*10^11 [1,0*10^10; 9,0*10^12], m = 5,1*10^12 (8,3*10^12)*	*N = 4, Me = 1,8*10^13 [3,0*10^12; 6,0*10^13], m = 4,1*10^13 (3,2*10^13)*	*N = 9, Me = 1,0*10^12 [2,0*10^11; 8,0*10^12], m = 4,6*10^12 (6,7*10^12)*	*0,502*	–	–	–
p6#1 сутки#Lactobacillus spp., КОЕ	*N = 14, Me = 1,0*10^5 [0; 2,0*10^5], m = 1,5*10^9 (5,3*10^9)*	*N = 4, Me = 1,0*10^5 [5,0*10^4; 2,1*10^6], m = 2,0*10^6 (1,1*10^6)*	*N = 9, Me = 8,0*10^5 [1,0*10^5; 3,0*10^7], m = 3,0*10^7 (6,5*10^7)*	*0,227*	–	–	–
p7#1 сутки#Bifidobacterium spp., КОЕ	*N = 14, Me = 3,0*10^8 [2,0*10^7; 8,0*10^8], m = 1,6*10^10 (5,3*10^10)*	*N = 4, Me = 2,8*10^10 [5,0*10^9; 1,3*10^11], m = 9,2*10^10 (6,5*10^10)*	*N = 9, Me = 6,0*10^9 [1,0*10^8; 1,0*10^10], m = 5,1*10^10 (1,3*10^11)*	*0,066*	–	–	–
p8#1 сутки#Escherichia coli, КОЕ	*N = 14, Me = 4,0*10^8 [6,0*10^7; 2,0*10^9], m = 3,6*10^9 (8,1*10^9)*	*N = 4, Me = 1,7*10^9 [2,5*10^8; 6,5*10^9], m = 4,6*10^9 (3,4*10^9)*	*N = 9, Me = 2,0*10^9 [7,0*10^6; 3,0*10^9], m = 4,1*10^9 (6,7*10^9)*	*0,765*	–	–	–
p9#1 сутки#Bacteroides spp., КОЕ	*N = 14, Me = 2,2*10^11 [4,0*10^9; 9,0*10^12], m = 5,1*10^12 (8,3*10^12)*	*N = 4, Me = 1,8*10^13 [3,0*10^12; 6,0*10^13], m = 4,1*10^13 (3,2*10^13)*	*N = 9, Me = 1,0*10^12 [8,0*10^10; 8,0*10^12], m = 4,5*10^12 (6,8*10^12)*	*0,378*	–	–	–
p10#1 сутки#Faecalibacterium prausnitzii, КОЕ	*N = 14, Me = 7,5*10^6 [3,0*10^5; 2,0*10^9], m = 8,0*10^9 (1,8*10^10)*	*N = 4, Me = 2,0*10^8 [5,5*10^7; 1,5*10^11], m = 1,5*10^11 (7,5*10^10)*	*N = 9, Me = 5,0*10^8 [9,0*10^7; 7,0*10^8], m = 2,4*10^11 (6,6*10^11)*	*0,414*	–	–	–
p11#1 сутки#Klebsiella pneumoniae, КОЕ	*N = 14, Me = 0 [0; 0], m = 1,2*10^5 (3,0*10^5)*	*N = 4, Me = 0 [0; 1,0*10^5], m = 1,0*10^5 (5,0*10^4)*	*N = 9, Me = 0 [0; 2,0*10^6], m = 4,6*10^7 (1,3*10^8)*	*0,604*	–	–	–

p12#1 сутки#Klebsiella oxytoca, КОЕ	*N = 14, Me = 0 [0; 0], m = 0 (0)*	*N = 4, Me = 0 [0; 1,0*10^6], m = 1,0*10^6 (5,0*10^5)*	*N = 9, Me = 0 [0; 0], m = 0 (0)*	0,056	–	–	–
p14#1 сутки#Staphylococcus aureus, КОЕ	*N = 14, Me = 0 [0; 0], m = 5,0*10^7 (1,0*10^8)*	*N = 4, Me = 1,4*10^8 [4,0*10^7; 1,0*10^14], m = 1,0*10^14 (5,0*10^13)*	*N = 9, Me = 0 [0; 0], m = 0 (0)*	0,016	0,092	0,705	0,012
p15#1 сутки#Escherichia coli enteropathogenic, КОЕ	*N = 14, Me = 0 [0; 0], m = 2,9*10^3 (1,1*10^4)*	*N = 4, Me = 0 [0; 0], m = 0 (0)*	*N = 9, Me = 0 [0; 0], m = 0 (0)*	0,629	–	–	–
p16#1 сутки#Enterococcus spp., КОЕ	*N = 14, Me = 5,0*10^4 [0; 2,0*10^5], m = 2,9*10^6 (1,1*10^7)*	*N = 4, Me = 5,0*10^4 [0; 1,0*10^5], m = 5,8*10^4 (5,0*10^4)*	*N = 9, Me = 1,0*10^5 [0; 1,0*10^5], m = 1,6*10^5 (2,4*10^5)*	0,869	–	–	–
p17#1 сутки#Bacteroides thetaiotaomicron, КОЕ	*N = 13, Me = 1,0*10^7 [0; 3,0*10^8], m = 3,9*10^8 (8,4*10^8)*	*N = 4, Me = 1,5*10^10 [1,0*10^8; 1,7*10^11], m = 1,5*10^11 (8,3*10^10)*	*N = 9, Me = 2,0*10^8 [0; 7,0*10^8], m = 7,8*10^8 (1,6*10^9)*	0,264	–	–	–
p18#1 сутки#Akkermansia muciniphila, КОЕ	*N = 14, Me = 0 [0; 0], m = 7,9*10^8 (2,7*10^9)*	*N = 4, Me = 0 [0; 0], m = 0 (0)*	*N = 9, Me = 0 [0; 0], m = 3,3*10^8 (1,0*10^9)*	0,602	–	–	–
p19#1 сутки#Clostridium difficile, КОЕ	*N = 14, Me = 0 [0; 3,0*10^6], m = 1,8*10^8 (5,4*10^8)*	*N = 4, Me = 0 [0; 2,0*10^7], m = 2,0*10^7 (1,0*10^7)*	*N = 9, Me = 0 [0; 1,0*10^6], m = 2,2*10^9 (6,7*10^9)*	0,961	–	–	–
p20#1 сутки#Clostridium perfringens, КОЕ	*N = 14, Me = 0 [0; 0], m = 1,9*10^5 (5,5*10^5)*	*N = 4, Me = 0 [0; 3,0*10^8], m = 3,0*10^8 (1,5*10^8)*	*N = 9, Me = 0 [0; 0], m = 0 (0)*	0,344	–	–	–
p21#1 сутки#Proteus spp., КОЕ	*N = 14, Me = 6,0*10^7 [0; 6,0*10^9], m = 6,1*10^10 (1,5*10^11)*	*N = 4, Me = 2,5*10^10 [3,5*10^8; 5,0*10^10], m = 2,9*10^10 (2,5*10^10)*	*N = 9, Me = 1,0*10^9 [0; 4,0*10^9], m = 6,1*10^10 (1,7*10^11)*	0,456	–	–	–
p22#1 сутки#Enterobacter spp. / Citrobacter spp., КОЕ	*N = 14, Me = 1,1*10^8 [0; 3,0*10^9], m = 4,3*10^9 (9,1*10^9)*	*N = 4, Me = 3,0*10^7 [5,0*10^6; 5,0*10^9], m = 5,0*10^9 (2,5*10^9)*	*N = 9, Me =0 [0;0], m = 5,8*10^6 (1,7*10^7)*	0,084	–	–	–

p23#1 сутки#Fusobacterium nucleatum, КОЕ	*N = 14, Me =0 [0;0], m = 2,4*10^6 (8,0*10^6)*	*N = 4, Me =0 [0;0], m =0 (0)*	*N = 9, Me =0 [0;0], m =0 (0)*	0,223	–	–	–
p24#1 сутки#Parvimonas micra, КОЕ	*N = 14, Me =0 [0;0], m = 7,9*10^6 (2,7*10^7)*	*N = 4, Me =0 [0;0], m =0 (0)*	*N = 9, Me =0 [0;0], m = 1,1*10^5 (3,3*10^5)*	0,527	–	–	–
p25#1 сутки#Отношение Bacteroides fragilis group/ Faecalibacterium prausnitzii	*N = 14, Me = 1,7*10^4 [8,0*10^1; 1,0*10^10], m = 2,9*10^9 (4,7*10^9)*	*N = 4, Me = 4,5*10^5 [1,5*10^5; 5,0*10^9], m = 5,0*10^9 (2,5*10^9)*	*N = 9, Me = 2,9*10^3 [4,0*10^1; 2,0*10^5], m = 1,1*10^9 (3,3*10^9)*	0,708	–	–	–
p13#1 сутки#Candida spp., КОЕ	*N = 14, Me =0 [0;0], m = 2,1*10^6 (5,8*10^6)*	*N = 4, Me =0 [0; 1,0*10^7], m = 1,0*10^7 (5,0*10^6)*	*N = 9, Me =0 [0;0], m = 1,1*10^5 (3,3*10^5)*	0,739	–	–	–
Days 14							
p26#14 сутки#Общая бактериальная масса, КОЕ	*N = 14, Me = 3,0*10^11 [7,0*10^9; 5,0*10^12], m = 3,8*10^12 (5,9*10^12)*	*N = 4, Me = 1,0*10^12 [1,0*10^10; 3,1*10^13], m = 3,0*10^13 (1,6*10^13)*	*N = 6, Me = 4,0*10^11 [9,0*10^10; 8,0*10^11], m = 7,8*10^11 (1,1*10^12)*	0,993	–	–	–
p27#14 сутки#Lactobacillus spp., КОЕ	*N = 14, Me = 1,0*10^5 [0; 1,0*10^5], m = 2,1*10^8 (8,0*10^8)*	*N = 4, Me = 5,0*10^4 [0; 1,0*10^5], m = 5,8*10^4 (5,0*10^4)*	*N = 6, Me = 1,0*10^5 [1,0*10^5; 1,0*10^5]*	0,561	–	–	–
p28#14 сутки#Bifidobacterium spp., КОЕ	*N = 14, Me = 3,5*10^8 [2,0*10^7; 2,0*10^9], m = 4,0*10^9 (1,1*10^10)*	*N = 4, Me = 3,0*10^8 [5,0*10^7; 5,0*10^10], m = 5,0*10^10 (2,5*10^10)*	*N = 6, Me = 1,0*10^10 [3,0*10^8; 3,0*10^10], m = 1,1*10^11 (2,4*10^11)*	0,248	–	–	–
p29#14 сутки#Escherichia coli, КОЕ	*N = 14, Me = 3,0*10^8 [2,0*10^7; 1,0*10^9], m = 1,9*10^9 (5,2*10^9)*	*N = 4, Me = 3,5*10^9 [1,5*10^9; 6,5*10^9], m = 3,7*10^9 (4,0*10^9)*	*N = 6, Me = 1,5*10^7 [1,0*10^7; 3,0*10^7], m = 5,1*10^8 (1,2*10^9)*	0,050	–	–	–
p30#14 сутки#Bacteroides spp., КОЕ	*N = 14, Me = 3,0*10^11 [1,0*10^9; 5,0*10^12], m = 3,8*10^12 (5,9*10^12)*	*N = 4, Me = 1,0*10^12 [1,0*10^10; 3,1*10^13], m = 3,0*10^13 (1,6*10^13)*	*N = 6, Me = 3,0*10^11 [6,0*10^9; 8,0*10^11], m = 7,3*10^11 (1,2*10^12)*	0,922	–	–	–

p31#14 сутки#Faecalibacterium prausnitzii, КОЕ	*N = 14, Me = 6,5*10^6 [0; 4,0*10^8], m = 6,6*10^8 (1,6*10^9)*	*N = 4, Me = 2,2*10^8 [1,5*10^7; 4,5*10^10], m = 4,5*10^10 (2,3*10^10)*	*N = 6, Me = 5,0*10^7 [0; 1,0*10^9], m = 1,3*10^11 (3,3*10^11)*	*0,534*	–	–	–
p32#14 сутки#Klebsiella pneumoniae, КОЕ	*N = 14, Me =0 [0;0], m = 1,4*10^5 (5,3*10^5)*	*N = 4, Me =0 [0; 1,5*10^6], m = 1,5*10^6 (7,5*10^5)*	*N = 6, Me =0 [0;0], m =0 (0)*	*0,345*	–	–	–
p33#14 сутки#Klebsiella oxytoca, КОЕ	*N = 14, Me =0 [0;0], m =0 (0)*	*N = 4, Me =0 [0;0], m =0 (0)*	*N = 6, Me =0 [0;0], m =0 (0)*	*1,000*	–	–	–
p34#14 сутки#Candida spp., КОЕ	*N = 14, Me =0 [0;0], m = 5,6*10^5 (1,9*10^6)*	*N = 4, Me = 1,5*10^7 [0; 4,0*10^7], m = 2,4*10^7 (2,0*10^7)*	*N = 6, Me =0 [0;0], m = 1,2*10^7 (2,9*10^7)*	*0,361*	–	–	–
p35#14 сутки#Staphylococcus aureus, КОЕ	*N = 14, Me =0 [0; 1,0*10^7], m = 1,4*10^9 (5,3*10^9)*	*N = 4, Me =0 [0; 1,5*10^7], m = 1,5*10^7 (7,5*10^6)*	*N = 6, Me =0 [0;0], m = 1,7*10^5 (4,1*10^5)*	*0,558*	–	–	–
p36#14 сутки#Escherichia coli enteropathogenic, КОЕ	*N = 14, Me =0 [0;0], m =0 (0)*	*N = 4, Me =0 [0;0], m =0 (0)*	*N = 6, Me =0 [0;0], m =0 (0)*	*1,000*	–	–	–
p37#14 сутки#Enterococcus spp., КОЕ	*N = 14, Me =0 [0; 1,0*10^5], m = 1,4*10^8 (5,3*10^8)*	*N = 4, Me = 5,0*10^4 [0; 1,0*10^5], m = 5,8*10^4 (5,0*10^4)*	*N = 6, Me = 5,0*10^4 [0; 2,0*10^5], m = 8,3*10^4 (9,8*10^4)*	*0,821*	–	–	–
p38#14 сутки#Bacteroides thetaiotaomicron, КОЕ	*N = 14, Me = 5,1*10^7 [0; 6,0*10^8], m = 3,3*10^8 (4,2*10^8)*	*N = 4, Me = 2,0*10^9 [0; 4,0*10^11], m = 4,0*10^11 (2,0*10^11)*	*N = 6, Me =0 [0; 5,0*10^5], m = 3,3*10^8 (8,2*10^8)*	*0,510*	–	–	–
p39#14 сутки#Akkermansia muciniphila, КОЕ	*N = 14, Me =0 [0;0], m = 2,2*10^8 (8,0*10^8)*	*N = 4, Me =0 [0; 1,0*10^9], m = 1,0*10^9 (5,0*10^8)*	*N = 6, Me =0 [0;0], m =0 (0)*	*0,456*	–	–	–
p40#14 сутки#Clostridium difficile, КОЕ	*N = 14, Me =0 [0;0], m = 8,6*10^7 (2,7*10^8)*	*N = 4, Me =0 [0;0], m =0 (0)*	*N = 6, Me =0 [0;0], m = 1,2*10^10 (2,9*10^10)*	*0,631*	–	–	–
p41#14 сутки#Clostridium perfringens, КОЕ	*N = 14, Me =0 [0;0], m =0 (0)*	*N = 4, Me =0 [0;0], m =0 (0)*	*N = 6, Me =0 [0;0], m =0 (0)*	*1,000*	–	–	–

p42#14 сутки#Proteus spp., КОЕ	*N = 14, Me = 1,0*10^9 [4,0*10^6; 5,0*10^9], m = 2,8*10^9 (3,5*10^9)*	*N = 4, Me = 5,0*10^10 [2,0*10^10; 1,3*10^11], m = 8,7*10^10 (7,5*10^10)*	*N = 6, Me = 7,5*10^8 [1,0*10^7; 8,0*10^9], m = 3,3*10^9 (4,5*10^9)*	*0,092*	–	–	–
p43#14 сутки#Enterobacter spp. / Citrobacter spp., КОЕ	*N = 14, Me = 4,0*10^6 [0; 1,0*10^9], m = 1,2*10^9 (2,5*10^9)*	*N = 4, Me = 6,5*10^9 [1,6*10^9; 1,5*10^10], m = 8,9*10^9 (8,3*10^9)*	*N = 6, Me = 1,0*10^6 [0; 3,0*10^7], m = 2,2*10^7 (4,0*10^7)*	*0,025*	*0,055*	*>0,999*	*0,029*
p44#14 сутки#Fusobacterium nucleatum, КОЕ	*N = 14, Me =0 [0;0], m =0 (0)*	*N = 4, Me =0 [0;0], m =0 (0)*	*N = 6, Me =0 [0;0], m =0 (0)*	*1,000*	–	–	–
p45#14 сутки#Parvimonas micra, КОЕ	*N = 14, Me =0 [0;0], m = 7,1*10^5 (2,7*10^6)*	*N = 4, Me =0 [0;0], m =0 (0)*	*N = 6, Me =0 [0;0], m =0 (0)*	*0,700*	–	–	–
p46#14 сутки#Отношение Bacteroides fragilis group/ Faecalibacterium prausnitzii	*N = 14, Me = 533333,5 [5000,0; 9999999999,0], m = 3571982564,6 (4972023370,6)*	*N = 4, Me = 2833,5 [334,5; 102500,0], m = 99080,0 (51417,3)*	*N = 6, Me = 9444,5 [500,0; 9999999999,0], m = 3333336565,1 (5163975290,8)*	*0,256*	–	–	–
